# Supplementary material for: The interactome of intact mitochondria by cross-linking mass spectrometry provides evidence for coexisting respiratory supercomplexes
Source: Mol Cell Proteomics. 2017 Dec 8;17(2):216–32. doi: 10.1074/mcp.RA117.000470 (PMC5795388; doi:10.1074/mcp.RA117.000470)
Supplement: Supplemental Data [file supp_RA117.000470_133922_0_supp_23978_fzffwf.zip › spectra_annotation/mito_DR_spectra_annotation/165-2-3-1-9-1.pdf]

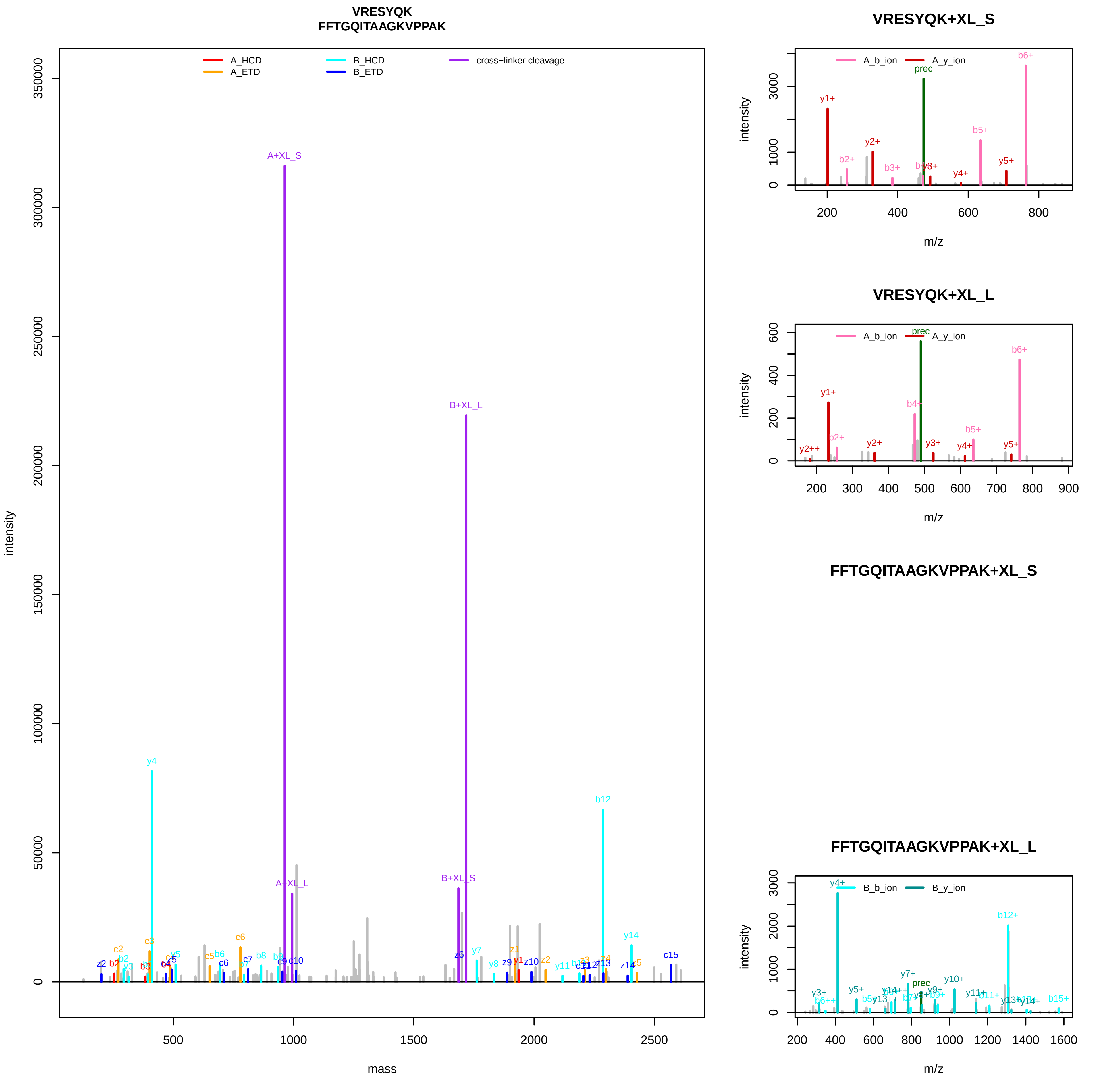
VRESYQK
FFTGQITAAGKVPPAK
A_HCD
A_ETD
B_HCD
B_ETD
cross−linker cleavage
A+XL_S
B+XL_L
A+XL_L
B+XL_S
y4
b12
y14
c15
intensity
mass
VRESYQK+XL_S
A_b_ion
A_y_ion
prec
y1+
y2+
b2+
b3+
b5+
b6+
y4+
y5+
m/z
VRESYQK+XL_L
y2++
b4+
y3+
FFTGQITAAGKVPPAK+XL_S
FFTGQITAAGKVPPAK+XL_L
B_b_ion
B_y_ion
y4+
b12+
y7+
y10+
y11+
b15+
